# Pathological Research

**Published:** 1920-10-09

**Authors:** 


### Pathological Research.

The necessity for independent pathological research in Australia owing to climatic and other differences, instead of relying upon work done in other parts of the world, has long been evident to earnest men here, and it is now proposed to establish the Institute of Pathological Research of New South Wales at the Royal North Shore Hospital (Sydney), and an appeal is now being made for a preliminary amount of £50,000 for the scheme, in which at least £150,000 will ultimately be required. The Scientific Advisory Council of the movement includes Professor D. A. Welsh (pathology), Dr. Henry Priestly (assistant professor of physiology), and Dr. E. W. Ferguson (acting principal microbiologist), all of the University of Sydney. Dr. A. H. Tebbutt, pathologist, of Macquarie Street, Sydney, and Dr. C. H. Burton Bradley, late director of microbiology for Queensland. Messrs. W. M. Vindin and Mr. Dugald Thomson are the Hon. Secretaries of the movement, and the offices are at 14 Castlereagh Street, Sydney, New South Wales.

It is announced that laboratory accommodation at the Institute will be at the disposal of any *bona-fide* scientist desirous of taking a definite line of research, and by the issue of reports and bulletins it is hoped to foster an Australian enthusiasm in medical matters. So far, we understand, the Government has not made any offer of help in this matter.

